# Political participation enhances subjective well-being in rural China: the dual mediating pathways of fairness and trust

**DOI:** 10.3389/fpsyg.2026.1858427

**Published:** 2026-07-16

**Authors:** Zhengang Zhang, Defei Wang

**Affiliations:** 1 School of Business Administration, South China University of Technology, Guangzhou, China; 2 School of Public Administration, South China University of Technology, Guangzhou, China

**Keywords:** political participation, rural residents, subjective well-being, social fairness perception, general social trust, subjective social status

## Abstract

**Background:**

Subjective well-being is a core indicator of livelihood quality and grassroots governance effectiveness. Against the backdrop of China’s Rural Revitalization Strategy, improving rural residents’ well-being is a central goal of national governance. However, existing scholarship has long focused on material determinants such as income and education, while lacking systematic analysis of the well-being transmission mechanisms of non-economic institutional factors like political participation. In particular, how grassroots political participation affects rural residents’ happiness through socio-cognitive pathways, and the heterogeneous boundary effects of subjective social status (SSS), remain critical research gaps in rural well-being studies.

**Methods:**

Drawing on Equity Theory and Social Cognitive Theory, this study constructs a moderated parallel mediation framework based on nationally representative data from the 2023 Chinese General Social Survey (CGSS). We employ OLS regression with robust standard errors, instrumental variable estimation, and bias-corrected Bootstrap testing to systematically examine the mechanisms and boundary conditions of village committee election participation on rural residents’ subjective well-being.

**Results:**

First, political participation exhibits a stable positive association with rural residents’ subjective well-being, which remains consistent across multiple robustness checks. However, instrumental variable estimation does not support definitive causal inference, so we prudently frame our core finding as a robust correlational association. Second, the association is transmitted through two parallel pathways: social fairness perception acts as the dominant mediating mechanism, while general social trust plays a secondary complementary role. Third, SSS exerts an asymmetric moderating effect: it only significantly strengthens the well-being returns of fairness perception among individuals with lower SSS, while no significant moderating effect is observed for the social trust pathway. Fourth, heterogeneity analysis shows that the well-being association is only significant among middle-aged (45–60 years) and high-income rural residents.

**Conclusion:**

This study delineates the dual psychological mechanisms and asymmetric stratification boundary of political participation’s well-being effects in rural China. It not only fills the academic gap of existing well-being research’s long-standing focus on material factors and neglect of non-economic psychological mechanisms, but also verifies the central role of institutional fairness perceptions in the well-being effects of grassroots governance. Our findings provide empirical evidence for advancing inclusive rural governance and precisely improving the sense of gain among different groups.

## 1 Introduction

As Hume noted, “The great end of all human industry is the attainment of happiness.” The pursuit of subjective well-being (SWB) is a core goal of human development and a central topic in positive psychology (Hume, 1777). The Report to the 20th National Congress of the Communist Party of China stresses that farmers form the grassroots foundation of national governance. Improving rural residents’ SWB is therefore critical for consolidating governance legitimacy, advancing rural revitalization, achieving common prosperity, and enhancing public trust in local authorities.

Since reform and opening up, China’s rural economy has grown rapidly, and material living standards have improved substantially. However, the Easterlin paradox has become increasingly evident in rural China: the marginal effect of economic growth on SWB has gradually weakened (Easterlin et al., 2010). Given the large rural population, exploring non-economic and psychological determinants of rural residents’ well-being has become an important academic task. As a key form of civic engagement and an indicator of grassroots democratic development, political participation has emerged as a critical non-economic factor worthy of rigorous investigation.

When rural residents participate in institutionalized political activities such as village elections and public deliberation, they are not merely exercising democratic rights but actively shaping their perceptions of the social environment. Extensive research confirms that social-cognitive evaluations play a foundational role in SWB formation (Layard, 2005; Stiglitz et al., 2009). The influence of political participation on well-being extends beyond mere behavioral involvement, residing instead in the specific psychological states it fosters. Social fairness perception—defined as an individual’s normative judgment regarding the equity of resource distribution, rule enforcement, and collective decision-making—is rooted in equity theory (Adams, 1965) and the organizational justice framework (Colquitt, 2001), with its core lying in assessments of procedural neutrality (Tyler, 1997). General social trust encapsulates a generalized expectancy in the predictability and benevolence of others and public institutions within the broader social order, classically captured by the item “most people can be trusted” (Rosenberg, 1956), and is regarded as a cornerstone of social cooperation (Yamagishi and Yamagishi, 1994). Both constructs exhibit robust empirical associations with SWB (Helliwell and Wang, 2011; Knack and Keefer, 1997) and serve as core variables for unpacking the psychological “black box” linking civic engagement to well-being.

However, three persistent limitations in the extant literature constrain a comprehensive understanding of this nexus. First, empirical investigations into the political participation–SWB link remain disproportionately concentrated on urban or nationally representative adult samples, resulting in the persistent marginalization of rural residents—a demographic constituting a formidable share of China’s populace and occupying a central position in national revitalization agendas. Second, while the dominant paradigm in well-being research continues to privilege economic determinants such as income, education, and housing (Veenhoven, 2005; Stevenson and Wolfers, 2008), it largely neglects the critical boundary condition of subjective social status (SSS). Derived from social comparisons, SSS is a well-documented moderator of socio-political returns (Anderson et al., 2012), yet its role in shaping the psychosocial dividends of civic engagement and recent evidence suggests its predictive power for psychological outcomes extends beyond conventional SES indicators such as education, occupation, and income (Robson et al., 2025) —yet its role in shaping the psychosocial dividends of civic engagement remains underexplored. remains underexplored. Finally, and most critically, there exists a profound theoretical void regarding the boundary conditions governing these mediated pathways. It remains unclear whether the indirect effects via these dual pathways are heterogeneous across levels of SSS, and no study has integrated these psychosocial mechanisms into a unified analytical framework to estimate their respective net associative effects.

To address these critical gaps, this study draws on nationally representative data from the 2023 Chinese General Social Survey (CGSS) to investigate three interconnected research questions:

1) The Main Linkage: What is the nature of the association between institutional political participation and rural residents’ subjective well-being (SWB), after accounting for potential endogeneity and confounding factors?
2) The Underlying Mechanisms: Do social fairness perception and general social trust act as parallel mediators in this relationship, and what is the relative strength of their respective indirect effects?
3) The Boundary Conditions: To what extent does subjective social status (SSS) moderate the strength of these two indirect pathways linking the mediators to SWB?

Addressing these questions systematically fills critical voids in the literature regarding rural sample coverage, the exploration of psychosocial mechanisms, and the identification of boundary conditions. It also provides empirically grounded insights to inform precision grassroots governance in the context of China’s rural revitalization initiative.

## 2 Theoretical framework and hypotheses

### 2.1 Political participation and subjective well-being

Extensive literature in political psychology and sociology has documented a positive association between various forms of civic engagement and subjective well-being (SWB). The core theoretical logic, rooted in self-determination theory, suggests that political participation fosters SWB by satisfying basic psychological needs for autonomy, competence, and relatedness. Through political behaviors such as self-expression, collective decision-making, and social integration, individuals can achieve stronger life satisfaction and positive affect (Ryan and Deci, 2000; Verba et al., 1995; Flavin and Keane, 2012). Empirical studies have consistently linked conventional political acts—from voting to community organizing—with higher SWB, while revealing meaningful heterogeneity across social contexts. For instance, Lorenzini (2015) identified systematic differences in the participation–well-being link between employed and unemployed European youth, supporting the view that the relationship is contextually shaped.

Against this theoretical background, it is necessary to examine whether the same pattern applies in rural China, which features a distinct institutional environment. Rural political participation in China is mainly embedded in the system of villagers’ self-governance, as legally defined in the Organic Law of the Villagers Committees. Formal participatory channels mainly include voting in village committee elections, attending villagers’ assemblies, deliberating on public affairs, and supervising village finances (National People’s Congress, 2018). These are not symbolic procedures but important mechanisms for rural residents to voice demands, affect the distribution of collective resources, and interact with grassroots governance (Zhang et al., 2004). Theoretically, such institutionalized participation can improve SWB through expressive, integrative, and instrumental functions: enhancing individual efficacy, strengthening community solidarity, and improving the quality of governance and resource allocation (Tsai, 2007).

Despite theoretical expectations, direct empirical evidence on how institutionalized political participation affects the holistic SWB of rural Chinese residents remains limited and inconclusive. Studies on rural governance have extensively examined village elections, social capital, and political trust, but seldom test their final impact on comprehensive SWB; most focus on intermediate outcomes such as political efficacy or policy satisfaction. For example, Dong and Kübler (2021) confirmed that government performance and political trust promote rural residents’ SWB but did not examine residents’ own participatory behaviors as a core explanatory factor. He (2023) found that farmers’ perceived social justice was positively associated with institutional participation, implying a potential psychological mechanism, yet did not verify the final link to SWB. Moreover, the direction of causality between participation and well-being remains debated in cross-national research. Some studies indicate that SWB may shape political participation rather than the reverse. For example, Lindholm (2020) observed that higher SWB reduced protest intentions in Switzerland, while Sulemana and Agyapong (2019) found no significant impact of life satisfaction on voting or protest behavior in Ghana. These mixed findings suggest that the participation–SWB relationship cannot be taken for granted in China’s rural context. Therefore, a clear research gap exists: it remains empirically unclear whether routine institutional political participation can significantly improve SWB among rural Chinese residents. Verifying this baseline relationship is essential for exploring subsequent mechanisms and boundary conditions. Accordingly, we propose:

*H1*: Political participation is positively associated with subjective well-being among rural Chinese residents.

### 2.2 The parallel mediating roles of social fairness perception and general social trust

To unpack the underlying psychological pathways linking political participation to subjective well-being (SWB), we draw on core insights from equity theory (Adams, 1965) and social cognitive perspectives (Bandura, 1986) to examine two parallel mediating pathways: social fairness perception and general social trust. These two social-cognitive constructs represent distinct yet complementary channels through which civic engagement may shape individual evaluations of life.

A substantial body of cross-cultural research validates both as robust psychological antecedents of happiness (Helliwell and Putnam, 2004). Social fairness perception, which centers on an individual‘s subjective judgment of procedural and distributive justice within society, is increasingly recognized as a more potent predictor of SWB than objective measures of inequality (Dufhues et al., 2026; Alesina et al., 2004). Concurrently, social trust, reflecting a generalized confidence in the integrity and cooperativeness of others, is a well-established pillar of social capital that strongly correlates with life satisfaction by reducing social uncertainty and facilitating cooperative gains (Putnam, 2000; Helliwell and Wang, 2011).

Prior research in the Chinese context has firmly established the centrality of these mediators. For instance, Hou et al. (2023) identify social fairness perception as a key conduit linking social capital to farmers’ political participation, while Ma et al. (2024) and Li and He (2022) demonstrate that governance quality and social security satisfaction flow through sequential pathways—fairness to trust—to enhance SWB. Extending this, Rothstein and Uslaner (2005) highlight the synergistic interplay between institutional fairness and social trust. However, conventional serial mediation models presuppose a rigid temporal hierarchy. In the milieu of rural China, these constructs are better understood as co-evolving and mutually constitutive rather than sequentially determined. To circumvent the imposition of arbitrary causal ordering and to isolate the net associative strength of each pathway, we employ a parallel mediation framework. This approach is particularly germane to rural governance, where institutional legitimacy (fairness) and interpersonal networks (trust) dynamically reinforce one another. By modeling these pathways concurrently, we mitigate the risk of effect-size obfuscation and offer a more granular account of how grassroots participation translates into well-being.

#### 2.2.1 The mediating role of perceived social fairness

Perceived social fairness refers to individuals’ subjective judgment of whether the rules and outcomes governing the distribution of social resources and opportunities are just. In the arena of rural governance, political participation provides residents with a direct channel to observe, intervene in, and legitimize the processes of village decision-making and public resource allocation. By participating in elections or village affairs meetings, residents gain insights into decision-making procedures, helping to ensure their interests are considered, thereby potentially enhancing their perceptions of both procedural and distributive fairness.

Substantial evidence indicates that fairness perception is a crucial antecedent of well-being. Research by Yang et al. (2025), based on national data, clearly demonstrates that perceived social fairness significantly enhances residents’ subjective well-being, with a stronger effect for those of lower socioeconomic status. The study by Li and He (2022) further reveals that satisfaction with social security boosts residents’ well-being through the sequential mediation of enhanced social fairness perception and social trust. This suggests that in the realm of public services, fairness perception is a key psychological link connecting institutional experiences with personal welfare. In the context of the COVID-19 pandemic, Wang and Zhang (2025) found that perceptions of social fairness (including opportunity and outcome fairness) enhanced residents’ sense of social security by increasing political trust, providing evidence for the protective psychological role of fairness perceptions during crises. Conversely, research by Yi and Li (2022) demonstrates from the opposite angle that income inequality, inadequate public goods provision, and corruption caused by inappropriate competition among local governments significantly reduce residents’ sense of fairness. This implies that grassroots political participation, if it can effectively monitor and correct such “inappropriate competition” behaviors, may serve as an important mechanism for maintaining and enhancing villagers’ sense of fairness. Based on the above logic, we hypothesize:

*H2a*: Perceived social fairness plays a positive mediating role in the relationship between political participation and the subjective well-being of rural residents.

#### 2.2.2 The mediating role of general social trust

General social trust, defined as a generalized confidence in other people and social institutions, is another robust predictor of well-being. High levels of social trust can reduce uncertainty in social interactions, promote cooperation and social support, thereby enhancing life satisfaction and emotional health (Helliwell and Wang, 2011). In closely-knit rural communities, political participation, as a form of collective action, provides an institutionalized, repetitive platform for interaction among villagers and between villagers and local cadres. This interaction helps reduce information asymmetry, establish norms of reciprocity, and thus foster generalized trust.

Recent research provides rich evidence for the multi-dimensional role of social trust in the Chinese context. A study by Chen and Zhu (2021) focusing on rural elderly found that trust in family, friends, and neighbors significantly improved their emotional well-being, with subjective well-being mediating this relationship. This indicates that “particularized trust” based on close circles is an important foundation for well-being. From a corporate governance perspective, research by Goodell et al. (2023) found that social trust at the regional level (macro trust) can act as an informal governance mechanism, influencing corporate behavior (e.g., reducing dividend payments to invest in innovation). This suggests that generalized trust at the village level (a form of meso-social capital) may also create a governance atmosphere that affects residents’ psychological experiences. Research by Lee and Ottervik (2024) in Latin America found that gender-equitable attitudes are associated with higher social and political trust, highlighting the link between social values and trust systems. In rural China, the process of inclusive and transparent political participation itself may be a process of disseminating equitable values and shaping a high-trust community culture. However, the relationship can be complex; research by Woelfert and Kunst (2020) on trust and social distancing compliance during the COVID-19 pandemic showed that the interaction between political and social trust is intricate, with high social trust potentially hindering policy compliance in contexts of low political trust. This suggests that when analyzing how political participation influences well-being through trust, the complex relationships between different types of trust must be considered. Nevertheless, overall, we expect that political participation helps build a positive social trust environment, thereby enhancing well-being. Therefore, we hypothesize:

*H2b*: General social trust plays a positive mediating role in the relationship between political participation and the subjective well-being of rural residents.

### 2.3 The moderating role of subjective social status (SSS)

Subjective social status (SSS)—an individual’s self-assessment of their position within the social hierarchy—integrates social comparison, lived experience, and affective evaluation. Compared to objective socioeconomic status indicators, SSS typically exhibits stronger predictive power for psychological and behavioral outcomes because it captures the experienced reality of inequality (Anderson et al., 2012). Importantly, SSS is not merely a reflection of objective class; it is a distinct dimension of stratification shaped by social context and perceived standing. In China’s transitional economy, for instance, SSS often diverges from objective indicators due to the influence of institutional transitions, rapid social change, and household-specific circumstances, highlighting its unique role in shaping life satisfaction (Zhao, 2012).

Regarding the social fairness perception pathway, fairness heuristic theory posits that individuals rely on fairness perceptions as a heuristic cue for environmental safety (Lind, 2001). However, the translation of this cue into well-being is contingent upon one’s relative status. Social comparison theory suggests that lower-SSS individuals exhibit heightened sensitivity to entitlement violations and relative deprivation (Smith et al., 2012). For these individuals, participation-induced gains in fairness represent a validation of rights, thereby substantially enhancing SWB (Tyler and Lind, 1992). Conversely, higher-SSS individuals often take procedural justice for granted and derive security primarily from resource advantages, leading to diminishing marginal utility of fairness perceptions on SWB (Anderson et al., 2012). Thus, SSS does not shape the initial formation of fairness perceptions from participation but fundamentally governs the terminal stage of resource translation—namely, it moderates the marginal utility of fairness perceptions in predicting SWB.

Turning to the general social trust pathway, trust in rural contexts functions primarily as “community infrastructure,” sustained by network structures and reciprocity norms rather than individual status appraisals (Putnam, 2000; Knack and Keefer, 1997). Unlike the evaluative nature of fairness, trust reflects stable expectations of repeated interactions within tight-knit communities (Yamagishi and Yamagishi, 1994). Research indicates that the returns on trust are largely determined by aggregate social capital stocks rather than individual rank anxiety (Coleman, 1990). Consequently, SSS is unlikely to penetrate the translation process from trust to SWB, and the effect size of this pathway is expected to remain invariant across SSS levels.

We therefore hypothesize:

*H3a*: SSS negatively moderates the strength of the association between perceived social fairness and SWB (i.e., the indirect effect is stronger for lower-SSS individuals).

*H3b*: SSS does not significantly moderate the strength of the association between general social trust and SWB.

*H4*: The overall indirect effect of political participation on SWB via the dual mediators is weaker for higher-SSS individuals, driven exclusively by the moderation of the fairness perception pathway.

**Figure 1:**
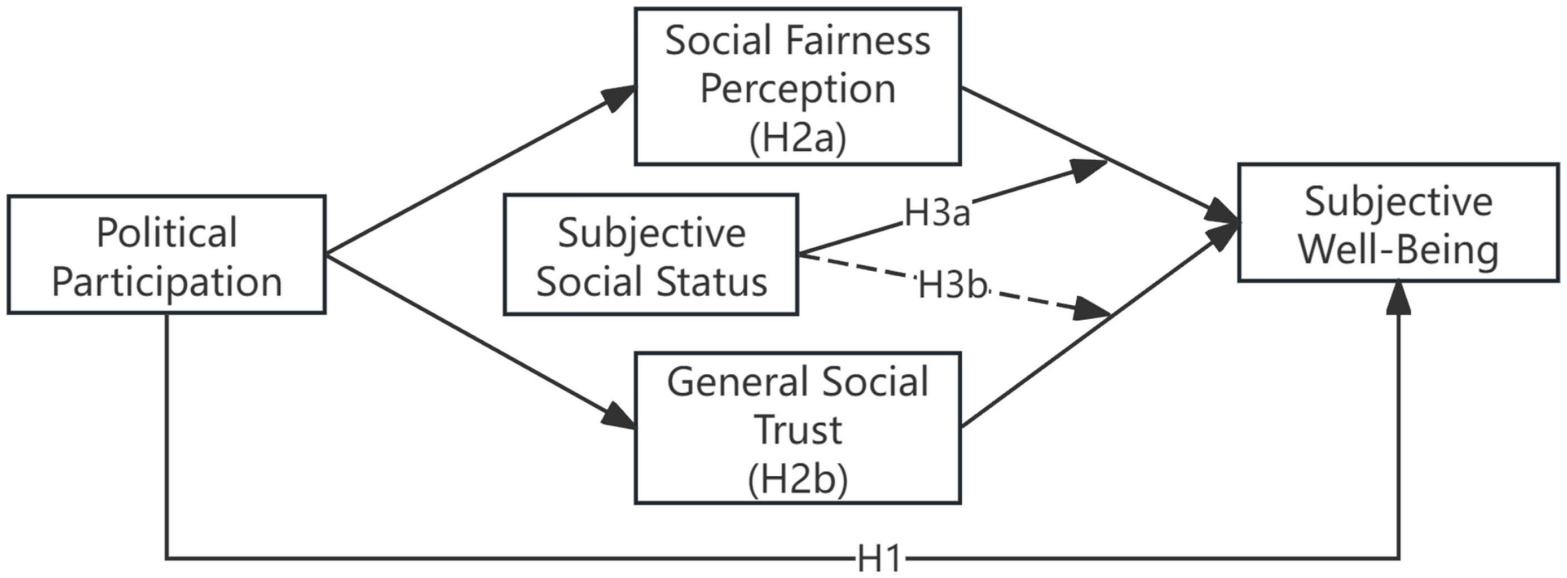
Conceptual framework. Hypothesis 3a (H3a) posits that subjective social status (SSS) negatively moderates the association between social fairness perception and subjective well-being (SWB). Hypothesis 3b (H3b) proposes that SSS exerts no statistically significant moderating effect on the association between general social trust and SWB; the moderating path for H3a is represented by a dashed arrow in the figure. Hypothesis 4 (H4) states that, driven exclusively by the moderation effect specified in H3a, the total indirect effect of political participation on SWB via the dual mediating pathways is weaker for individuals with higher subjective social status.

The research framework of this study is illustrated in Figure 1.

## 3 Research design

This section details the research design, data source, variable measurement, and empirical strategy to ensure the replicability and rigor of the study.

### 3.1 Data source and sample screening

#### 3.1.1 Data source

This study uses microdata from the 2023 China General Social Survey (CGSS) (2023), a nationally representative, cross-sectional social survey project. The CGSS is implemented by the National Survey Research Center at Renmin University of China. It employs a multi-stage stratified probability sampling method, covering 31 provinces, autonomous regions, and municipalities directly under the Central Government across mainland China (excluding Hong Kong, Macao, and Taiwan). It collects comprehensive data on individuals’ socioeconomic backgrounds, behavioral patterns, and social attitudes. The scientific rigor of the sampling design is well-documented (Bian and Li, 2012), and the specific measurements and implementation procedures for the 2023 wave are detailed in the survey’s technical documentation (Chinese General Social Survey (CGSS), 2023).

#### 3.1.2 Sample screening

To focus on the research object and ensure data quality, the sample is screened through four strict steps:

First, rural sample identification. Rural residents are identified according to the “household registration type” indicator in the questionnaire, where rural household registration = 1 and urban household registration = 0. Only respondents with rural household registration are retained, and urban samples are excluded.

Second, age restriction. To ensure respondents have legitimate voting rights and can reasonably and independently exercise political participation, only respondents aged 18 and above and below 70 are included in the final analysis.

Third, missing value treatment. Observations with missing information on core variables—including political participation, subjective well-being, social fairness perception, general social trust, and subjective social status (SSS)—are eliminated to avoid estimation bias.

Fourth, outlier and illogical response exclusion. Extreme values in continuous variables (e.g., household income) and illogical or contradictory answers are dropped to ensure data reliability.

After the above screening procedures, a total of 3,005 valid samples are retained for the empirical analysis. The sample size is sufficiently large to provide adequate statistical power for testing the proposed moderated mediation model.

### 3.2 Variable measurement

Combined with the variable coding rules in the CGSS 2023 codebook, the measurement methods of core variables and control variables in this study are specified as follows:

#### 3.2.1 Dependent variable: subjective well-being (SWB)

Measured using the question “Overall, do you feel your life is happy?” (variable code: a36) from the CGSS questionnaire.

Coding Rule: 5-point ordinal scale, where 1 = “very unhappy,” 2 = “unhappy,” 3 = “neutral,” 4 = “happy,” 5 = “very happy.”

Description: Higher values indicate higher subjective well-being of rural residents.

#### 3.2.2 Independent variable: political participation

Measured using the question “Did you Vote in the last neighborhood committee/village committee election?” (variable code: a44) from the CGSS questionnaire.

Coding Rule: Binary variable, where 1 = “Yes, participated in voting,” 0 = “No, did not participate in voting” (Note: Responses of “Not eligible / Not participating in the election / Not aware” are excluded or recoded as missing, consistent with sample screening rules).

Description: A value of 1 indicates that the rural resident has engaged in formal grassroots political participation behavior.

#### 3.2.3 Mediating variables

(1) Social Fairness Perception

Measured using the question “In general, do you think the current social distribution is fair?” (variable code: a35).

Coding Rule: 5-point ordinal scale, where 1 = “very unfair,” 2 = “unfair,” 3 = “neutral,” 4 = “fair,” 5 = “very fair.”

Description: Higher values reflect stronger subjective perception of social fairness among rural residents.

(2) General Social Trust

Measured using the question “How much do you trust most people in society?” (variable code: a33).

Coding Rule: 5-point ordinal scale, where 1 = “completely distrust,” 2 = “distrust,” 3 = “neutral,” 4 = “trust,” 5 = “completely trust.”

Description: Higher values represent higher levels of general social trust among rural residents.

#### 3.2.4 Moderating variable: subjective social status (SSS)

Measured using the question: “In our society, some individuals occupy the upper echelons while others reside at the lower levels. Overall, within the current social structure, which tier do you personally belong to?” (variable code: a43a). Simultaneously, it undergoes centralization processing to avoid multicollinearity issues in subsequent regression analyses.

Coding Rule: 10-point ordinal scale, with values ranging from 1 (lowest subjective social status) to 10 (highest subjective social status).

Description: The variable directly reflects rural residents’ self-perceived relative position in the social hierarchy.

#### 3.2.5 Control variables

To eliminate the interference of confounding factors, the following individual and family characteristic variables are controlled, consistent with the CGSS 2023 coding standards. The specific results are shown in Table 1.

**Table 1:** Control variable.

Variable name	Definition and measurement	Coding rule
Gender	Individual gender attribute	1 = male, 0 = female
Age	Actual age of the respondent in years	Continuous variable; age squared included to control non-linear relationship with SWB
Education Level	Highest educational attainment of the respondent	1 = Primary school and below (original codes 1–3) 2 = Junior high school (original code 4) 3 = High school/technical secondary/technical school (original codes 5–8) 4 = College and above (original codes 9–13) (Original code 14 = other is excluded or merged as appropriate)
Marital status	Marital status of the respondent	1 = married, 0 = unmarried/divorced/widowed
Party membership	Communist Party membership status	1 = Communist Party member, 0 = non-member
Household income (log)	Logarithm of total annual household income	Continuous variable (natural logarithm used to reduce skewness)

SWB, subjective well-being.

### 3.3 Empirical strategy

#### 3.3.1 Benchmark regression model

To examine the association between political participation and rural residents’ SWB (H1), we specify an OLS regression model with heteroskedasticity-robust standard errors:


$${\mathrm{SWB}}_{i}=\alpha_{0}+\alpha_{1}{\text{vote}}_{i}+\sum \alpha_{k}{\text{Control}}_{\mathrm{ik}}+\varepsilon_{i}$$ (1)

This Equation 1 is used to investigate the association between political participation and rural residents’ subjective well-being (H1). Among them,

${\mathrm{SWB}}_{i}$ = Subjective well-being of rural resident i;

${\text{vote}}_{i}$ = Core independent variable (political participation) of rural resident i;

${\text{Control}}_{\mathrm{ik}}$ = The *k*-th control variable;

$\alpha_{0}$ = Constant term; $\alpha_{1}$ and $\alpha_{k}$ = Regression coefficients to be estimated;

$\varepsilon_{i}$ = Random error term.

Robust standard errors are used to address potential heteroscedasticity, ensuring the reliability of coefficient significance.

#### 3.3.2 Parallel mediating effect test

To verify the parallel mediating roles of social fairness perception and general social trust (H2a and H2b), Hayes’ Process Macro Model 4 (Hayes, 2013) is adopted, with 5,000 Bootstrap repetitions to test the significance of mediating effects. The model specifications are:

1 First-stage regression (mediators):

$${\text{Fairness}}_{i}=\beta_{0}+\beta_{1}{\text{vote}}_{i}+\sum \beta_{k}{\text{Control}}_{\mathrm{ik}}+\mu_{i}$$ (2)

$${\text{Trust}}_{i}=\gamma_{0}+\gamma_{1}{\text{vote}}_{i}+\sum \gamma_{k}{\text{Control}}_{\mathrm{ik}}+\nu_{i}$$ (3)

2 Second-stage regression (dependent variable):

$$\begin{array}{c} {\mathrm{SWB}}_{i}=\delta_{0}+\delta_{1}{\text{vote}}_{i}+\delta_{2}{\text{Fairness}}_{i}+\delta_{3}{\text{Trust}}_{i}\\ +\sum \delta_{k}{\text{Control}}_{\mathrm{ik}}+\omega_{i} \end{array}$$ (4)

Equations 2–4:

${\text{Fairness}}_{i}$ = Social fairness perception of rural resident i

${\text{Trust}}_{i}$ = General social trust of rural resident i

${\mathrm{SWB}}_{i}$ = Subjective well-being of rural resident i;

${\text{vote}}_{i}$ = Core independent variable (political participation) of rural resident i;

${\text{Control}}_{\mathrm{ik}}$ = The *k*-th control variable;

$\mu_{i}$ $\nu_{i}\;\omega_{i}$ = Random error terms.

A mediating effect is considered statistically significant if the 95% bootstrap confidence interval (CI) for the indirect effect does not contain 0.

#### 3.3.3 Moderated parallel mediating effect test

To test the moderating role of subjective social status (SSS) in the direct path and two mediating paths (H3a, H3b and H4), Hayes’ Process Macro Model 14 (Hayes, 2013) is used. The detailed specifications are provided in Equations 5–7.

First-stage regression (mediators):


$$\text{Fairnes}s_{i}=\beta_{0}+\beta_{1}\mathrm{vot}e_{i}+\beta_{2}\mathrm{SS}S_{i}+\sum \beta_{k}\text{Contro}l_{\mathrm{ik}}+\mu_{i}$$ (5)

$$\begin{array}{c} \begin{array}{c} \text{Trus}t_{i}=\gamma_{0}+\gamma_{1}\mathrm{vot}e_{i}+\gamma_{2}\mathrm{SS}S_{i}+\sum \gamma_{k}\text{Contro}l_{\mathrm{ik}}+\nu_{i} \end{array} \end{array}$$ (6)

Second-stage regression (dependent variable):


$$\begin{array}{c} \mathrm{SW}B_{i}=\lambda_{0}+\lambda_{1}\mathrm{vot}e_{i}+\lambda_{2}\text{Fairnes}s_{i}+\lambda_{3}(\text{Fairnes}s_{i}\times \mathrm{SS}S_{i})+\lambda_{4}\text{Trus}t_{i}\\ +\lambda_{5}(\text{Trus}t_{i}\times \mathrm{SS}S_{i})+\lambda_{6}\mathrm{SS}S_{i}+\sum \lambda_{k}\text{Contro}l_{\mathrm{ik}}+\omega_{i} \end{array}$$ (7)

in Equation 7.

1) Interactions between social fairness perception and SSS ( $\text{fairnes}s_{i}\times \mathrm{SS}S_{i}$ )
2) Interactions between general social trust and SSS ( $\text{trus}t_{i}\times \mathrm{SS}S_{i}$ )

The significance of the moderating effect is determined by the coefficient of the interaction term and the 95% bootstrap confidence interval. If the CI does not include 0, the moderating effect is statistically significant.

To further illustrate the pattern of moderation, simple slope analysis is conducted at three levels of SSS:

Low SSS (mean − 1 SD)

Mean SSS

High SSS (mean + 1 SD)

This procedure clarifies how the strength of the direct and indirect associations varies across different levels of subjective social status.

## 4 Empirical results

This section presents the empirical findings based on 3,005 valid observations of rural residents drawn from the 2023 Chinese General Social Survey (CGSS). All regression models employ heteroskedasticity-robust standard errors to ensure estimation reliability. The analysis proceeds sequentially from descriptive statistics and baseline regression, to endogeneity tests and robustness checks to validate result stability, followed by heterogeneity analysis to explore subgroup variations, and finally examines mediating mechanisms and moderated mediation effects to uncover the underlying psychological pathways and boundary conditions.

### 4.1 Descriptive statistics and multicollinearity test

#### 4.1.1 Descriptive statistics

Table 2 reports the descriptive statistics of all core variables, providing a foundational overview of the sample characteristics. The dependent variable, subjective well-being (SWB), has a mean value of 3.858 (SD = 0.862) on a 5-point scale, indicating that rural residents in the sample report a moderate-to-high level of life satisfaction.

**Table 2:** Descriptive statistics of core and control variables.

Variable	Mean	SD	Min	Max
Political participation (Vote = 1)	0.538	0.500	0	1
Social fairness perception (Fairness)	3.300	1.036	1	5
General social trust (Trust)	3.496	1.049	1	5
Subjective social status (SSS)	4.125	1.878	1	10
Subjective well-being (SWB)	3.858	0.862	1	5
Age	3.853	0.320	2.890	4.248
Gender (male = 1)	0.470	0.500	0	1
Education level (education)	2.090	1.047	1	4
Party membership (party_member)	0.093	0.291	0	1
Marital status (marry)	0.802	0.399	0	1
Household income	10.599	1.170	7.601	13.122
Observations	3,005	3,005	3,005	3,005

SWB, Subjective Well-Being; SSS, Subjective Social Status. For binary variables, the mean represents the proportion.

The core independent variable, political participation (measured by participation in village committee elections), has a mean of 0.538 (SD = 0.500), meaning 53.8% of rural residents participated in the most recent grassroots election, reflecting a moderate level of institutionalized political engagement in rural China.

For the mediating variables: social fairness perception has a mean of 3.300 (SD = 1.036), and general social trust has a mean of 3.496 (SD = 1.049), both falling in the medium range of their respective 5-point scales. The moderating variable, subjective social status (SSS), has a mean of 4.125 (SD = 1.878) on a 10-point scale (1 = lowest, 10 = highest), indicating substantial variation in rural residents’ self-perceived social standing.

Among control variables: 47.0% of the sample is male (Gender = 0.470); the average education level is 2.090 (SD = 1.047) on a 4-point scale (1 = primary school and below, 4 = college and above); 9.3% are Communist Party members (Party Membership = 0.093); 80.2% are married (Marital Status = 0.802); and the mean household income is 10.599 (SD = 1.170). The analysis is based on N = 3,005 valid observations after data cleaning.

#### 4.1.2 Multicollinearity test

To evaluate potential multicollinearity among key variables—including political participation, social fairness perception, general social trust, subjective social status, their interaction term, and the age-squared term—we conduct a Variance Inflation Factor (VIF) test using the full baseline regression specification.

First, to mitigate multicollinearity between age and its squared term, we center the age variable around its sample mean before constructing the quadratic term. This transformation effectively eliminates the collinearity between the linear and quadratic age terms, which is a common source of inflated VIF values in nonlinear models.

Table 3 reports the VIF values for all variables included in the regression. As shown, all VIF values are below 2, with a mean VIF of only 1.30—far below the conventional threshold of 10 (and the stricter threshold of 5 recommended for psychological research). Specifically, the VIF values range from 1.01 (for the interaction term Vote×SSS) to 1.81 (for education level), with other core variables such as social trust (VIF = 1.17) and social fairness perception (VIF = 1.18) exhibiting minimal collinearity.

**Table 3:** Variance inflation factor (VIF) test results.

Variable	VIF	1/VIF
Education level (edu)	1.81	0.552
Centered age (c_age)	1.79	0.557
Centered age squared (c_age_sq)	1.44	0.694
Log household income (log_income)	1.43	0.701
Marital status (marry)	1.29	0.773
Party membership (party_member)	1.19	0.837
Social fairness perception (fairness)	1.18	0.845
General social trust (trust)	1.17	0.852
Political participation (std_vote)	1.13	0.885
Subjective social status (std_SSS)	1.09	0.915
Gender	1.04	0.960
Interaction term (std_interact)	1.01	0.991
Mean VIF	1.30	–

VIF, Variance Inflation Factor. Values greater than 10 indicate severe multicollinearity, while values below 5 are generally acceptable. The mean VIF is calculated across all predictors.

This result confirms that multicollinearity is not a concern in our regression analyses. The inclusion of the interaction term and the quadratic age term does not introduce severe collinearity issues, ensuring the stability and reliability of the coefficient estimates in subsequent analyses.

### 4.2 Baseline regression results: direct association between political participation and SWB

To address Research Question 1 (RQ1)—the nature of the association between institutional political participation and SWB—and to formally test Hypothesis 1 (H1), we employ stepwise OLS regression with heteroskedasticity-robust standard errors. Table 4 reports the full results.

**Table 4:** Stepwise baseline regression results.

Variable	(1) Core variables only	(2) Add controls	(3) Add fairness	(4) Add trust	(5) Add both mediators	(6) Add SSS
Political participation (vote)	0.090^***^ (0.033)	0.121^***^ (0.034)	0.074^**^ (0.033)	0.098^***^ (0.034)	0.068^**^ (0.033)	0.053 (0.032)
Social fairness perception (fairness)	_	_	0.240^***^ (0.017)	_	0.219^***^ (0.018)	0.193^***^ (0.017)
General social trust (trust)	_	_	_	0.135^***^ (0.017)	0.063^***^ (0.017)	0.059^***^ (0.016)
SSS	_	_	_	_	_	0.171^***^ (0.041)
Fairness*SSS	_	_	_	_	_	−0.025^***^ (0.009)
Trust*SSS	_	_	_	_	_	−0.011 (0.010)
Age (centered)	_	−0.001 (0.002)	−0.002 (0.002)	−0.003^*^ (0.002)	−0.003^*^ (0.002)	−0.003^*^ (0.002)
Age squared (centered)	_	0.000*** (0.000)	0.000*** (0.000)	0.000*** (0.000)	0.000*** (0.000)	0.000*** (0.000)
Education level (2 = Junior high)	_	−0.007 (0.041)	0.022 (0.039)	0.003 (0.041)	0.024 (0.039)	0.017 (0.039)
Education level (3 = High school/technical)	_	0.096* (0.049)	0.087* (0.047)	0.073 (0.049)	0.077 (0.047)	0.0787* (0.046)
Education level (4 = College and above)	_	0.135** (0.060)	0.122** (0.059)	0.100* (0.060)	0.107* (0.059)	0.108* (0.058)
Gender	_	−0.033 (0.032)	−0.047 (0.030)	−0.037 (0.031)	−0.048 (0.030)	−0.020 (0.030)
Party_member	_	0.165*** (0.049)	0.153*** (0.047)	0.152*** (0.048)	0.148*** (0.047)	0.125*** (0.046)
Marry	_	0.268*** (0.048)	0.274*** (0.046)	0.56*** (0.048)	0.267*** (0.046)	0.262*** (0.045)
Household income (Log)	_	0.123*** (0.018)	0.116*** (0.017)	0.122*** (0.018)	0.116*** (0.017)	0.0975*** (0.017)
Region fixed effects	Controlled	Controlled	Controlled	Controlled	Controlled	Controlled
Constant	4.042^***^ (0.071)	2.178^***^ (0.224)	1.546^***^ (0.217)	1.758^***^ (0.223)	1.406^***^ (0.219)	1.674^***^ (0.216)
Observations	3,005	3,005	3,005	3,005	3,005	3,005
*R* ^2^	0.021	0.085	0.165	0.110	0.170	0.196

Standard errors are in parentheses. Significance levels: ***p < 0.01, **p < 0.05, *p < 0.10. Education level: 1, Primary, 2, Junior high, 3, High school/technical, 4, College and above. Reference groups: 1.edu (Primary school and below), 0.sex (female), 0.party_member (non-Party member), 0.marry (unmarried). Coefficients are rounded to three decimal places for consistency.

Model (1) only examines the direct effect of political participation on rural residents’ subjective well-being, without including any control variables. The coefficient of political participation is 0.090 and statistically significant at the 1% level, indicating a significantly positive raw association between political participation and SWB.

Model (2) adds a series of control variables to further examine the effect of political participation on SWB. After controlling for gender, age, age squared, education, Communist Party membership, marital status, log household income, and region fixed effects, the coefficient of political participation is 0.121 and remains highly significant at the 1% level. This result demonstrates that political participation still exerts a significant and positive effect on SWB after accounting for confounding factors, providing robust empirical support for H1. The explanatory power of the model improves substantially, with *R*^2^ rising from 0.012 to 0.085.

Models (3) and (4) (Add Mediators Separately) reveal that the direct association between political participation and SWB weakens when each mediator is included: Model 3 (Add Fairness) shows the coefficient of political participation drops to 0.074 (*p* < 0.05), while social fairness perception is highly significant ( β = 0.240, *p* < 0.01); Model 4 (Add Trust) shows the coefficient of political participation increases to 0.098 (*p* < 0.01), with general social trust also significant ( β = 0.135, *p* < 0.01). This suggests that both variables may act as mediating channels.

Model (5) (Add Both Mediators) confirms this: the direct coefficient of political participation becomes statistically insignificant ( β = 0.068, *p* < 0.05), while both social fairness perception and general social trust remain significant. This indicates that the positive association between political participation and SWB is largely transmitted through these two parallel mediating pathways—foreshadowing the formal mediating effect analysis below.

Finally, Model (6) incorporates subjective social status (SSS) and its interaction terms with social fairness perception and general social trust, to test the hypothesized moderating effects on the second half of the mediating pathways. Results show that SSS exerts a significant positive direct effect on SWB. Specifically, the interaction term between social fairness perception and SSS is significantly negative ( β = −0.025, *p* < 0.01), indicating that SSS negatively moderates the association between perceived social fairness and SWB (the second stage of the fairness mediating pathway). The interaction term between general social trust and SSS is statistically non-significant ( β = −0.011, *p* > 0.10). These findings confirm the partial mediating roles of social fairness perception and general social trust, and provide preliminary empirical support for the subsequent formal moderated mediation analysis.

In summary, the independent positive association between political participation and SWB remains robust across all model specifications, thereby confirming H1.

### 4.3 Endogeneity test: instrumental variable (IV) estimation

To further clarify the nature of the association identified in RQ1 and to address potential endogeneity concerns (e.g., reverse causality or omitted variable bias), we conduct a Two-Stage Least Squares (2SLS) estimation. While the baseline OLS results confirm a robust association, the IV approach helps assess the feasibility of a causal interpretation.

#### 4.3.1 Instrumental variable selection

We use the county-level voting rate as the instrumental variable. This variable reflects the overall level of political participation at the county level, which strongly predicts an individual’s likelihood of participating in village elections but should not directly affect individual subjective well-being through channels other than political participation. This instrument therefore satisfies both the relevance and exogeneity conditions required for valid IV estimation.

#### 4.3.2 2SLS estimation results

Table 5 reports the IV estimation results. Column (1) presents the first-stage regression, where the county-level voting rate is strongly and positively associated with individual political participation ( β = 0.927, *p* < 0.001). The first-stage F-statistic is 79.519 well above the critical value of 10, confirming no weak instrument problem and validating the relevance of the IV.

**Table 5:** Endogeneity test: IV 2SLS results.

Variable	(1) First stage: political participation (Vote)	(2) Second stage: SWB
County-level voting rate (county_vote_rate)	0.927^***^ (0.054)	–
Political participation (vote)	–	0.025 (0.122)
Age (centered)	0.007*** (0.001)	−0.003 (0.003)
Age squared (centered)	−0.000*** (0.000)	0.001*** (0.000)
Education level (2 = Junior high)	0.072** (0.027)	−0.006 (0.059)
Education level (3 = High school/technical)	0.122*** (0.039)	0.022 (0.079)
Education level (4 = College and above)	0.079 (0.058)	0.204** (0.101)
Gender	0.031 (0.023)	−0.010 (0.048)
Party_member	0.232*** (0.033)	0.176* (0.093)
Marry	0.083** (0.034)	0.411*** (0.078)
Household income (Log)	0.002 (0.011)	0.130*** (0.024)
Constant	−0.098 (0.126)	2.011*** (0.277)
Observations	1,478	1,478
First-stage *F*-statistic	79.519	–
*R* ^2^	0.243	–
Second-stage Wald *χ*^2^	–	53.504

Robust standard errors in parentheses. Column (1) reports first-stage regression results with vote as the dependent variable. Column (2) reports second-stage 2SLS results with happiness as the dependent variable. Standard errors are in parentheses. Significance levels: ***p < 0.01, **p < 0.05, *p < 0.10. Reference groups: 1.edu (Primary school and below), 0.sex (Female), 0.party_member (Non-Party member), 0.marry (Unmarried). The first-stage *F*-statistic is manually calculated to test for weak instruments.

In the second-stage regression (Column 2), the estimated coefficient of political participation on subjective well-being is 0.025 (SE = 0.122), which is statistically insignificant, reflecting the lower efficiency of IV estimation relative to OLS. Meanwhile, the second-stage Wald χ^2^ is 53.504 (*p* < 0.001), indicating that the overall model is highly statistically significant.

In conclusion, while the baseline OLS results robustly support H1, the IV estimation fails to confirm a causal relationship. Consequently, we characterize political participation as a correlational contributor to SWB rather than a deterministic causal driver for the remainder of the analysis, consistent with the limitations of cross-sectional survey data.

### 4.4 Robustness checks

To verify the stability of the baseline association between political participation and SWB, We conducted two robustness checks; the results, which consistently support the baseline conclusions, are presented in Table 6.

**Table 6:** Robustness check results.

Variable	(1) Binary outcome Probit	(2) Winsorized NOLS
Political participation (vote)	0.047*** (0.016)	0.122*** (0.032)
Age (centered)	−0.001 (0.001)	−0.001 (0.002)
Age squared (centered)	0.000*** (0.000)	0.000*** (0.000)
Education level (2 = Junior high)	−0.010 (0.020)	−0.000 (0.041)
Education level (3 = High school/technical)	0.038 (0.025)	0.089* (0.048)
Education level (4 = College and above)	0.061** (0.029)	0.134** (0.057)
Gender	−0.022 (0.016)	−0.0314 (0.0309)
Party_member	0.097*** (0.022)	0.167*** (0.049)
Marry	0.147*** (0.023)	0.286*** (0.048)
Household income (Log)	0.050*** (0.008)	0.116*** (0.017)
Region fixed effects	Controlled	Controlled
Constant	0.034 (0.085)	2.219*** (0.183)
Observations	3,005	3,005
*R* ^2^	0.065	0.072

Robust standard errors are in parentheses. Column (1) reports average marginal effects from a probit model; Column (2) reports OLS estimates with log household income winsorized at the 1st and 99th percentiles. Standard errors are in parentheses. Significance levels: ***p < 0.01, **p < 0.05, *p < 0.10. Reference groups: 1.edu (Primary school and below); 0.sex (Female); 0.party_member (Non-party member); 0.marry (Unmarried).

#### 4.4.1 Alternative outcome measurement

We redefine the dependent variable as a binary indicator of “high well-being” (happiness_bin), coded as 1 if the original SWB score is ≥ 4 (happy or very happy) and 0 otherwise. We then estimate a probit regression with the same set of control variables. Column (Adams, 1965) of Table 6 shows that the coefficient of political participation remains positive and highly significant ( β = 0.047, *p* < 0.001), confirming that the positive association is not dependent on the ordinal measurement of SWB.

#### 4.4.2 Outlier mitigation

To address potential bias from extreme values in the continuous control variable (logged household income), we winsorize the variable at the 1st and 99th percentiles (to limit the influence of extreme high or low income observations) and re-run the OLS regression. Column (2) of Table 6 demonstrates that the coefficient of political participation remains positive and significant ( β = 0.122, *p* < 0.001), with similar magnitude to the baseline result. This confirms that outliers do not drive the observed association.

Overall, the results from both robustness checks consistently support the baseline conclusion: political participation is positively and robustly associated with subjective well-being among rural residents.

### 4.5 Heterogeneity analysis

To explore whether the association between political participation and SWB varies across subgroups, we conduct heterogeneity analysis by age and household income—two key demographic and economic characteristics that may shape rural residents’ political engagement and well-being.

#### 4.5.1 Age heterogeneity

We divide the sample into three age groups: young (< 45 years), middle-aged (45–60 years), and elderly (> 60 years). Table 7 reports the subgroup results:

**Table 7:** Heterogeneity analysis results.

Variable	(1) Young (< 45)	(2) Middle-aged (45–60)	(3) Elderly (> 60)	(4) Low income	(5) High income
Political participation (vote)	0.127 (0.091)	0.197*** (0.073)	−0.042 (0.105)	0.120 (0.081)	0.114* (0.063)
Controls	Yes	Yes	Yes	Yes	Yes
Region fixed effects	Controlled	Controlled	Controlled	Controlled	Controlled
Observations	370	725	383	714	764
*R* ^2^	0.131	0.115	0.041	0.060	0.087

Robust standard errors in parentheses. Standard errors are in parentheses. Significance levels: ***p < 0.01, **p < 0.05, *p < 0.10. All regressions include the full set of control variables (centered age, centered age squared, education level, gender, party membership, marital status, and logged household income).

For young rural residents, the coefficient of political participation (vote) is 0.127 (*p* > 0.1), statistically insignificant.

For middle-aged rural residents, the coefficient is 0.197 (*p* < 0.01), positive and highly significant—the strongest association among all age groups.

For elderly rural residents, the coefficient is −0.042 (*p* > 0.1), statistically insignificant and directionally negative.

This pattern suggests that the positive association is concentrated among middle-aged working-age rural residents, who may have more direct stakes in village governance outcomes (e.g., resource allocation, public service provision) and thus derive greater psychological benefits from political participation. Younger residents may still be establishing their social and economic footing, while elderly residents’ well-being may be more driven by health and family support, reducing the salience of political participation.

#### 4.5.2 Income heterogeneity

We split the sample into low-income and high-income groups based on the median of logged household income. The results of the heterogeneity analyses based on household income are shown in Table 7.

For low-income rural residents, the coefficient of political participation is 0.120 ( p > 0.1), statistically insignificant.

For high-income rural residents, the coefficient was 0.114 ( SE=0.063,p<0.1) , which was positive and marginally significant.

This indicates that the positive association is more pronounced among high-income rural residents, who may have greater capacity to engage meaningfully in political activities (e.g., accessing information, advocating for interests) and thus derive more tangible psychological benefits from participation.

Notably, these heterogeneous patterns reflect differences in the strength of the cross-sectional correlation, not causal effects, consistent with our overall analytical framework.

### 4.6 Mediating effect analysis: parallel pathways of fairness and trust

To formally test H2a and H2b (the parallel mediating roles of social fairness perception and general social trust in the association between political participation and SWB), we adopt a two-stage ordinary least squares (OLS) regression approach with heteroskedasticity-robust standard errors. Detailed regression results are presented in Table 8.

**Table 8:** Mediating effect analysis: parallel pathways of fairness and trust.

Variable	(1) Fairness path first stage (Dep. Var.: fairness)	(2) Fairness path second stage (Dep. Var.: SWB)	(3) Trust path first stage (Dep. Var.: trust)	(4) Trust path second stage (Dep. Var.: SWB)
Political participation (vote)	0.195*** (0.042)	0.074** (0.033)	0.165*** (0.042)	0.098*** (0.034)
Social fairness perception (fairness)	–	0.240*** (0.017)	–	–
General social trust (trust)	–	–	–	0.135*** (0.017)
Age (centered)	0.004* (0.002)	−0.002 (0.002)	0.011*** (0.002)	−0.003* (0.002)
Age squared (centered)	−0.000 (0.000)	0.000*** (0.000)	0.000*** (0.000)	0.000*** (0.000)
Education level (2 = junior high)	−0.121** (0.051)	0.022 (0.039)	−0.070 (0.050)	0.003 (0.041)
Education Level (3 = high school/technical)	0.039 (0.062)	0.087* (0.047)	0.170*** (0.061)	0.073 (0.049)
Education Level (4 = college and above)	0.057** (0.076)	0.122** (0.059)	0.258*** (0.081)	0.100* (0.060)
Gender	0.060 (0.039)	−0.047 (0.030)	0.030 (0.039)	−0.037 (0.031)
Party_member	0.0508 (0.067)	0.153*** (0.047)	0.102 (0.066)	0.152*** (0.048)
Marry	−0.0216 (0.054)	0.274*** (0.046)	0.091* (0.055)	0.256*** (0.048)
Household income (Log)	0.0308 (0.021)	0.116*** (0.017)	0.004 (0.020)	0.122*** (0.018)
Region fixed effects	Controlled	Controlled	Controlled	Controlled
Constant	2.638*** (0.259)	1.546*** (0.217)	3.716*** (0.215)	1.758*** (0.223)
Observations	3,005	3,005	3,005	3,005
*R* ^2^	0.041	0.165	0.062	0.110

Standard errors are in parentheses. Significance levels: ***p < 0.01, **p < 0.05, *p < 0.10.

In the fairness pathway, political participation significantly and positively predicted social fairness perception ( β = 0.195, *p* < 0.001). In turn, fairness perception positively predicted SWB ( β = 0.240, *p* < 0.001), while the coefficient of political participation weakened from 0.121 to 0.074 (*p* < 0.05). The indirect effect was 0.0468, supporting the mediating role of fairness perception and confirming H2a. In the trust pathway, political participation positively predicted general social trust ( β = 0.165, *p* < 0.001). Trust then positively predicted SWB ( β = 0.135, *p* < 0.001), and the political participation coefficient decreased from 0.121 to 0.098 (*p* < 0.01). The indirect effect was 0.0223, supporting the mediating role of trust and confirming H2b. Overall, the two parallel pathways produced a total indirect effect of 0.0691, accounting for 57.1% of the total effect. The fairness pathway contributed 67.7% of the total indirect effect, while the trust pathway contributed 32.3%. These findings demonstrate that political participation enhances rural residents’ SWB through two distinct and parallel psychological pathways, with the fairness perception pathway playing a dominant role.

Collectively, these results provide robust empirical validation for both H2a and H2b. Political participation enhances rural residents’ SWB through two distinct and parallel psychological channels, with the fairness perception pathway serving as the principal mechanism.

### 4.7 Moderating effect analysis: the role of subjective social status (SSS)

Having established the dual mediating pathways in RQ2—where social fairness perception emerged as the dominant mechanism—we next formally test the moderating role of subjective social status (SSS) to address Research Question 3 (RQ3: To what extent does SSS moderate the strength of the two indirect pathways linking political participation to SWB). This analysis evaluates Hypotheses H3a, H3b, and H4, employing a bias-corrected nonparametric percentile bootstrap procedure with 5,000 resamples to estimate conditional indirect effects at three SSS levels: low (1 SD below the mean), medium (mean), and high (1 SD above the mean). Detailed statistical results are reported in Table 9.

**Table 9:** Conditional indirect effects by SSS level (Bootstrap, 5,000 repetitions).

Pathway	SSS level	Coefficient	Standard error	95% confidence interval
Political Participation → Social Fairness Perception → SWB	Low (Mean − 1 SD)	0.068*	0.015	[0.041, 0.099]
	Medium (Mean)	0.049*	0.009	[0.032, 0.068]
	High (Mean + 1 SD)	0.029*	0.012	[0.009, 0.053]
	Low vs. High Difference	0.039*	0.016	[0.012, 0.071]
Political Participation → General Social Trust → SWB	Low (Mean − 1 SD)	0.013**	0.006	[0.002, 0.024]
	Medium (Mean)	0.011**	0.005	[0.003, 0.022]
	High (Mean + 1 SD)	0.010	0.005	[−0.002, 0.021]

Bootstrap replications = 5,000; seed = 12,345. ***p < 0.01, **p < 0.05, *p < 0.10 (two-tailed). CI, confidence interval; SSS, subjective social status; SWB, subjective well-being.

For the social fairness perception pathway, the conditional indirect effect is 0.068 (SE = 0.015, 95% CI [0.041, 0.099]) at low SSS, 0.049 (SE = 0.009, 95% CI [0.032, 0.068]) at medium SSS, and 0.029 (SE = 0.012, 95% CI [0.009, 0.053]) at high SSS. All effects are statistically significant, and the magnitude of the effect decreases monotonically with increasing SSS. The difference in indirect effects between the low and high SSS groups is 0.039 (SE = 0.016, 95% CI [0.012, 0.071]), with the confidence interval excluding zero. These results directly support H3a, which posits that SSS negatively moderates the association between perceived social fairness and SWB, yielding stronger indirect effects for lower-SSS individuals. This pattern aligns with our theoretical prediction: as an evaluative construct heavily reliant on relative social judgment, the marginal well-being gains derived from perceived fairness diminish as individuals’ subjective social standing rises.

For the general social trust pathway, the conditional indirect effect is 0.013 (SE = 0.006, 95% CI [0.002, 0.024]) at low SSS and 0.011 (SE = 0.005, 95% CI [0.003, 0.022]) at medium SSS, both reaching marginal statistical significance. At high SSS, the effect decreases to 0.010 (SE = 0.005, 95% CI [−0.002, 0.021]) and becomes statistically non-significant. The difference in indirect effects between the low and high SSS groups is 0.002 (SE = 0.005, 95% CI [−0.009, 0.014]), with the confidence interval including zero. These findings confirm H3b, which predicts that SSS exerts no significant moderating effect on the association between general social trust and SWB. This is consistent with the characterization of generalized trust as “community infrastructure”: rooted in rural social networks and reciprocity norms, the efficiency of translating generalized trust into well-being remains relatively invariant across SSS levels.

The total indirect effect of political participation on SWB via the dual mediators declines significantly with increasing SSS, and this heterogeneity is entirely driven by the moderating effect observed in the social fairness perception pathway; the magnitude of the general social trust pathway does not differ significantly across SSS levels. This pattern fully validates H4, which holds that the total indirect effect is weaker among individuals with higher SSS, with heterogeneity entirely attributable to the moderation of the fairness perception pathway.

Collectively, the empirical results provide full support for all proposed hypotheses targeting RQ3. SSS exerts asymmetric moderating effects—selectively attenuating the fairness perception pathway while leaving the trust pathway unaffected—which clarifies the core boundary conditions of the political participation–SWB linkage in rural China.

## 5 Conclusion and practical implications

### 5.1 Conclusion

This study investigates how political participation shapes the subjective well-being (SWB) of rural residents, systematically addressing the three research questions posed in the Introduction. The core findings are summarized as follows:

First, regarding Research Question 1 (The Main Linkage): This study confirms a robust positive association between institutional political participation and rural residents’SWB, supporting H1. Notably, instrumental variable tests failed to establish a stable causal link, suggesting that political participation functions primarily as a correlational contributor to well-being rather than a deterministic causal factor. Future research should explore more rigorous identification strategies to disentangle this relationship.

Second, regarding Research Question 2 (Underlying Mechanisms): The results validate the parallel mediating roles of social fairness perception and general social trust, lending support to H2a and H2b. Crucially, social fairness perception is identified as the dominant mediating pathway, exhibiting a significantly larger effect size than general social trust. This finding addresses the prevailing gap in the literature that tends to prioritize economic determinants over psychological mechanisms.

Third, regarding Research Question 3 (Boundary Conditions): The analysis confirms the asymmetric moderating effect of subjective social status (SSS). SSS significantly attenuates the strength of the association between social fairness perception and SWB (H3a supported), whereas it exerts no significant moderating effect on the pathway linking general social trust to SWB (H3b supported). Furthermore, the total indirect effect of political participation on SWB weakens significantly as SSS increases, validating H4. These results clarify the stratified heterogeneity of the psychological dividends derived from political participation and refine the applicability boundaries of classic theories within the context of rural China.

### 5.2 Practical implications

First, optimize institutionalized participation channels and deliver targeted empowerment for groups with low subjective social status (SSS). In light of our finding that individuals with lower SSS derive significantly greater well-being gains from social fairness perception, policy design should go beyond periodic village committee elections to institutionalize diversified participation channels including village deliberation councils, public affairs supervision, and digital village affairs disclosure. Meanwhile, capacity-building training for political participation should be provided for disadvantaged groups to lower the institutional threshold for expressing demands, ensuring that the psychological dividends of political participation are precisely delivered to the groups most in need of support.

Second, anchor the core role of procedural justice and design differentiated stratified support policies. Consistent with the finding that social fairness perception acts as the dominant mediating pathway, grassroots governance should prioritize both procedural justice and distributive justice: priority should be given to improving the transparency of resource allocation for infrastructure investment and public service provision, and a closed-loop demand response and correction mechanism should be established. For high-SSS groups who exhibit lower sensitivity to well-being gains from fairness perception, general social trust can be fostered through community building, neighborhood mutual assistance and related activities, to improve their subjective well-being through complementary channels.

Third, implement a full-life-cycle differentiated strategy to match the heterogeneous needs across subgroups. For the middle-aged group (45–60 years old), for whom the well-being association of political participation is most pronounced, participation mechanisms should be deeply integrated with their core livelihood needs, including rural entrepreneurship support, local development planning, and public facility construction. For young residents, political participation agendas should be linked to integrated urban–rural employment and vocational skills training to enhance the relevance of participation. For elderly residents, participation content should be aligned with old-age security and primary medical service provision to address their core concerns. For low-income groups, for whom the participation effect is statistically insignificant, participation barriers should be reduced by simplifying procedures and lowering information acquisition costs, supplemented by targeted resource tilting to improve their participation capacity.

Fourth, build a multi-dimensional collaborative governance system to avoid over reliance on a single policy instrument. It should be clarified that political participation is a correlational factor rather than a panacea for improving rural well-being. Participatory governance should be advanced in coordination with inclusive public policies covering income security, healthcare, and educational resource allocation, to construct an integrated support ecosystem of “participation empowerment and livelihood guarantee” and maximize rural residents’ sense of gain and happiness.

## 6 Discussion

### 6.1 Theoretical contributions

First, this study breaks through the materialist paradigm that has long dominated rural well-being research by integrating non-economic psychological factors into the core explanatory framework. Existing scholarship has predominantly focused on economic determinants such as income and educational attainment. Building on Dolan et al. (2008)‘s foundational finding regarding the association between civic participation and well-being, this study further unpacks the underlying psychological mechanisms linking the two. By integrating Equity Theory (Adams, 1965) and Social Cognitive Theory (Bandura, 1986), we shift the analytical perspective from material resource endowments to “psychological architecture,” establishing political participation as a critical non-economic correlate of well-being and expanding the application scope of positive psychology in rural development research.

Second, this study clarifies the relative roles of parallel mediating pathways and identifies the dominant role of justice perceptions in the participation-well-being linkage. Distinct from the limitations of prior single-mediator models (He, 2023; Li and He, 2022), we employ a parallel mediation framework to disentangle the distinct contributions of different psychological channels. We find that social fairness perception serves as the dominant mediating mechanism, which strongly corroborates the core proposition of Equity Theory that justice perceptions are a fundamental source of subjective well-being (Adams, 1965), and aligns with Helliwell and Wang (2011)‘s conclusion that collective action fosters reciprocal norms. This finding contrasts with Rothstein and Uslaner (2005)‘s classic “fairness breeds trust” thesis. Combined with Huhe et al. (2015)‘s research on rural social trust in China, we demonstrate that in the context of China’s rural differential mode of association (the standard academic translation of Fei Xiaotong’s core concept), institution-based fairness perceptions have stronger explanatory power for well-being than acquaintance-based generalized social trust.

Third, this study verifies the asymmetric moderating effect of subjective social status (SSS) and revises the universal applicability assumption of social cognitive theory. As one of the first studies to apply a moderated mediation framework in the Chinese rural context, we find that SSS only selectively attenuates the second stage of the fairness mediating pathway (i.e., the link from fairness perception to SWB), while exerting no significant moderating effect on either the first stage of the fairness pathway (political participation → fairness perception) or any stage of the general social trust pathway. This finding is consistent with Social Comparison Theory (Anderson et al., 2012), indicating that individuals with lower SSS are significantly more sensitive to institutional justice. Furthermore, the null moderating effect of SSS on the trust pathway differs from the findings of Lee and Ottervik (2024). Combined with Freitag and Traunmüller (2009)‘s research on face-to-face interaction mechanisms, we show that particularized trust rooted in kinship and geographic ties in rural China is relatively independent of individual status perceptions, thereby revising the prevailing universal assumption regarding the boundary conditions of status signal effects.

### 6.2 Limitations and future research directions

Despite its theoretical and practical contributions, this study has several limitations that point to meaningful directions for future research:

Cross-sectional data limitation: This study relies on cross-sectional data from the 2023 CGSS, which limits definitive causal identification even after instrumental variable estimation. Future studies could use panel data to track rural residents over time or adopt quasi-experimental designs to better identify causal relationships between political participation and subjective well-being.

Measurement of political participation: Political participation is operationalized using a single indicator (village committee election voting), which may not fully capture the diversity of rural civic engagement (e.g., deliberation, supervision, community volunteering, and informal participation). Future research could employ multi-item measurement scales to capture different forms and intensities of political participation and compare their distinct well-being implications.

Unobserved confounding factors: Although this study controls for a comprehensive set of individual, household, and regional characteristics, some unobserved variables (such as personality traits, political efficacy, and local governance norms) could still shape the results. Future studies may incorporate more psychological and contextual variables to reduce potential omitted variable bias.

Additional moderators and mediators: This study focuses on subjective social status as a moderator and social fairness perception and general social trust as mediators. Future research could explore other important mechanisms, such as political efficacy, sense of belonging, or governance satisfaction, and examine potential moderators including regional institutional quality, village collective economy, and digital governance coverage.

Exploration of Structural Equation Modeling (SEM): While the “stepwise regression and Bootstrap” approach (Hayes’ PROCESS Macro) employed in this study offers high transparency and is well-suited for models with manifest variables, it is limited in its ability to account for measurement error. Future research equipped with multi-item scales could construct latent variables for core constructs (e.g., social fairness perception, general social trust, and subjective well-being) and utilize SEM for a more comprehensive test. SEM would allow for the simultaneous estimation of measurement and structural models, providing a more refined validation of the psychological mechanisms underlying political participation.

Contextual and comparative research: This study focuses on rural China. Future research could conduct systematic comparisons between rural and urban areas or across national contexts to clarify how institutional, cultural, and developmental factors shape the political participation–well-being linkage and its underlying psychological mechanisms.

Overall, this study provides a systematic empirical analysis of the association between political participation and subjective well-being among rural residents, along with its mediating and moderating mechanisms. Addressing the above limitations will further strengthen causal identification, measurement precision, and theoretical generalizability, ultimately supporting more targeted policies for rural governance, revitalization, and well-being enhancement.

## Data Availability

Publicly available datasets were analyzed in this study. This data can be found here: the dataset analyzed in this study is publicly available from the official repository of the China General Social Survey (CGSS). The raw data supporting the conclusions of this article can be freely accessed and downloaded upon registration from the China National Survey Data Archive (CNSSDA) at: http://www.cnsda.org. The data used corresponds to the 2023 wave (CGSS 2023) of the survey, which is the 13th nationally representative, cross-sectional survey conducted by the National Survey Research Center at Renmin University of China.
